# Assessment of the Effect of Four Kneeling Chair Angle Combinations on Muscle Activity and Perceived Discomfort

**DOI:** 10.3390/s26030970

**Published:** 2026-02-02

**Authors:** Xiaoxiao Lei, Jutao Li, Jingchen Cong, Mengyang Ren, Zhongxia Xiang

**Affiliations:** 1 School of Mechanical Engineering, Tianjin University, Tianjin 300354, China; lei_xiao2@tju.edu.cn (X.L.); xiangzhx@tju.edu.cn (Z.X.); 2 Key Laboratory of Mechanism Theory and Equipment Design of Ministry of Education, Tianjin University, Tianjin 300354, China; 3 School of Architecture & Art Design, Hebei University of Technology, Tianjin 300131, China; congjingchen@hebut.edu.cn; 4 Department of Industrial and Systems Engineering, The Hong Kong Polytechnic University, Hong Kong 999077, China; mengyang.ren@connect.polyu.hk

**Keywords:** kneeling chair, angle combination, surface electromyography, discomfort

## Abstract

The kneeling chair has an advantage over the traditional chair in that it promotes lumbar lordosis, while findings regarding muscle activity and subjective (dis)comfort evaluation between the two are inconsistent. Furthermore, there is a lack of studies focusing on the angle combinations of the kneeling chair. This study investigated an unsupported traditional sitting configuration and four unsupported kneeling configurations with different angle combinations through measurements of surface electromyography (EMG) signals, body part discomfort (BPD) scores, and seat configuration evaluation scores. A significant decrease in lumbar erector spinae (LES) muscle activity and perceived discomfort was observed in the kneeling configuration compared with the results for the traditional sitting configuration. Among the four angle combinations, the optimal arrangement was identified, which resulted in significantly less LES muscle activity and perceived discomfort. Our findings provide guidance for the design and future studies of the kneeling chair.

## 1. Introduction

Currently, sitting at work is very common. A study of 40 workstations found that 45% of office workers maintained a bent and unsupported back [1], which can result in sustained stretch of passive lumbar structures and extremely low levels of lower back muscle activity and thus, possible exacerbation of lower back pain [2]. Ergonomic adjustments of the workstation, consisting of the desk, chair, and other equipment, can improve work-related posture and reduce lower back pain [3]. The chair adjustment is easier and more feasible than the desk adjustment and can improve musculoskeletal symptoms among sedentary workers [4]. The normal lumbar curvature is maintained by a trunk–thigh angle of about 135 degrees; however, the lumbar curvature flattens as the trunk–thigh angle is reduced when using the traditional chair, thus causing lower back pain [5]. The chair with a forward-inclined seat pan can improve this situation [6]. The kneeling chair consists of a forward-inclined seat pan and a backward-inclined knee rest, and the kneeling posture has been found to be a closer approximation to the standing lumbar curvature than the traditional sitting posture [7,8]. Moreover, across different subject groups, postures, and tasks, lumbar lordosis can be better preserved when sitting in the kneeling chair than in the traditional chair [9,10]. A previous study [11] has summarized that reduced lumbar lordosis is one of frequently cited risk factors of occupational lower back pain. From this perspective, the kneeling chair shows great potential for workplace use.

Studies on the kneeling chair have been conducted based on surface electromyography (EMG) and subjective (dis)comfort evaluation, primarily focusing on comparing it with the traditional chair. Prior work is presented in Table 1. However, these comparisons have yielded inconsistent results. Considering the influence of the chair backrest on paraspinal muscle activity [12] and subjective (dis)comfort [13], as well as the absence of prior studies on the kneeling chair backrest, this study focuses on unsupported postures to eliminate potential effects of the backrest. In this regard, in the comparison between the unsupported traditional sitting posture and the unsupported kneeling posture, the findings of Soderberg et al. [14] and Wang et al. [15] are inconsistent.

Studies on the seat pan angle and the knee rest angle of the kneeling chair, both of which directly affect the kneeling posture, are limited. Through EMG measurement and comfort evaluation, Soderberg et al. [14] investigated the effect of seat pan angle on healthy adults, and Tang et al. [20] investigated the effect of seat pan angle and the effect of knee rest angle on healthy children; however, no prior studies have explored the effect of angle combination (i.e., the combination of seat pan angle and knee rest angle) on healthy adults.

## 2. Materials and Methods

### 2.1. Subjects

A total of 17 right-handed subjects, including 9 males and 8 females, participated in the experiment. None of the subjects had experienced any neck, back, or lower limb disorders for at least three months. None of the subjects had experience in using the kneeling chair. The subjects were aged from 19 to 30 years (M = 22.82, SD = 2.81), with the height ranging from 1.58 to 1.78 m (M = 1.68, SD = 0.06) and the body mass index (BMI) less than 28 kg/m^2^. Each subject signed an informed consent form before the experiment.

### 2.2. Experimental Design

This study investigated five seat configurations: seat configuration level 1 (SCL1) represents the unsupported traditional sitting configuration, and SCL2 to SCL5 represent four unsupported kneeling configurations with different angle combinations. Seat pan angles of 10 degrees (SA10) and 20 degrees (SA20) were set in the experiment, with reference to previous research investigating different seat pan angles in adults [14]. Knee rest angles of 20 degrees (KA20) [10,17] and 35 degrees (KA35) [21] were selected according to prior research on the kneeling chair that reported specific knee rest angles, since few previous studies have investigated different knee rest angles in adults. The angle combinations of SCL2 to SCL5 were SA10–KA20, SA10–KA35, SA20–KA20, and SA20–KA35, respectively.

**Figure 1:**
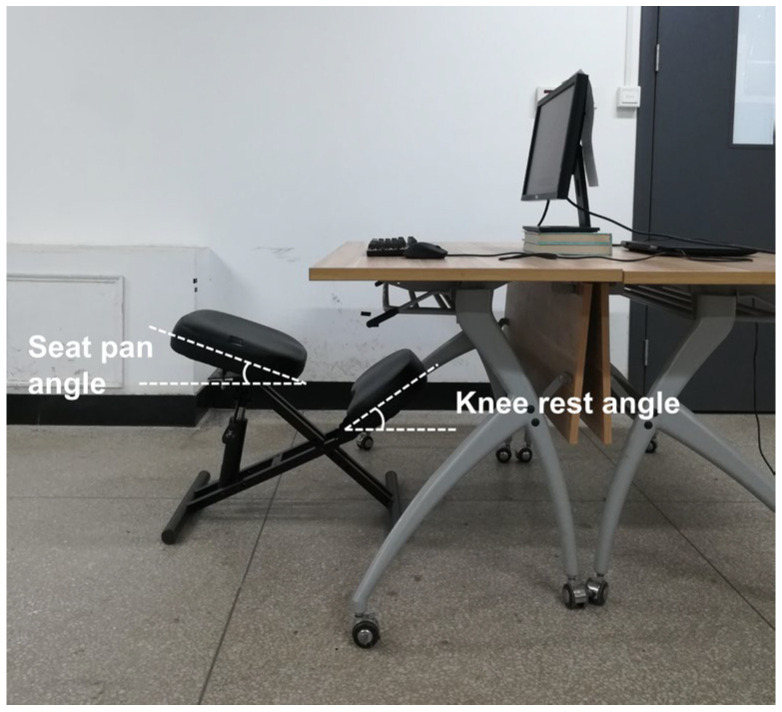
Experimental equipment.

As shown in Figure 1, the experimental equipment consisted of a kneeling chair (Anji Youdemeng Furniture Trading Co., Ltd., Huzhou, China), an HP Omen laptop (HP Inc., Shanghai, China), a Dell E2210H monitor (Dell Inc., Xiamen, China), a Rapoo V500pro keyboard (Shenzhen Rapoo Technology Co., Ltd., Shenzhen, China), and a Logitech M280 mouse (Logitech Inc., Suzhou, China). The desk height was approximately 74 cm. The monitor was positioned at a distance of approximately 70 cm from the subjects, and its top was level with subjects’ eyes [22]. The keyboard was placed approximately 10 cm from the edge of the desk, and its typing area was centered relative to the monitor. The mouse position could be adjusted by the subjects, and the keyboard position was not adjustable.

**Figure 2:**
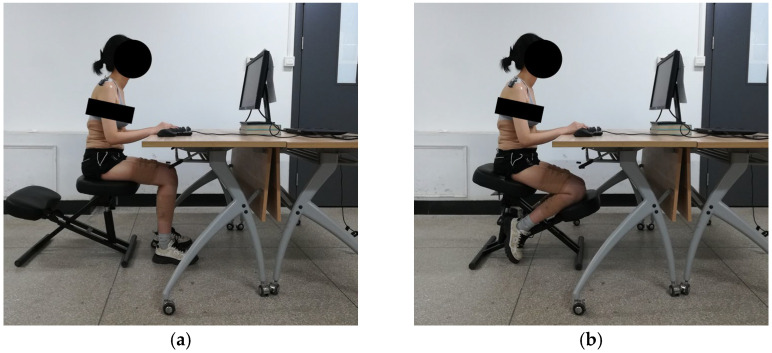
Experimental scenarios of (**a**) the traditional sitting posture and (**b**) the kneeling posture.

The experimental scenarios of the traditional sitting posture and kneeling posture are shown in Figure 2. The traditional chair was replaced with the kneeling chair to eliminate potential effects of factors such as seat pan material. In the kneeling posture, the subjects were instructed to place their lower legs on the knee rest, with their weight supported by both the seat pan and the knee rest. In the traditional sitting posture, the subjects were required to keep approximately 90 degrees of knee flexion with both feet on the ground [23].

**Figure 3:**
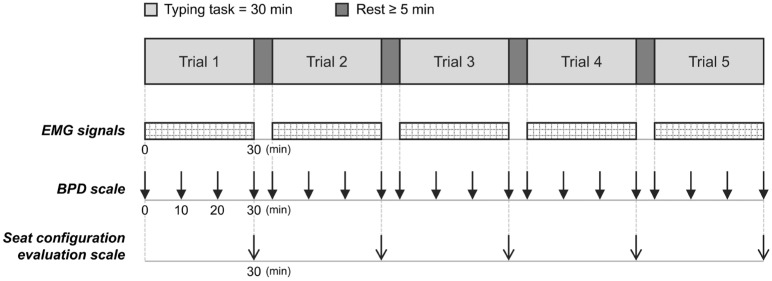
Experimental protocol. The EMG signals were collected continuously. The BPD scale and seat configuration evaluation scale were administered at specific time points indicated by arrows (EMG, surface electromyography; BPD, body part discomfort).

The experimental protocol was explained to the subjects in detail before the experiment. During this period, they maintained the kneeling posture to become familiar with using the kneeling chair. As shown in Figure 3, each subject completed five trials. Before each trial, the subjects were required to adopt the most comfortable posture possible and to try to maintain this posture without unnecessary postural adjustments or speech during the trial [17]. The subjects performed a 30 min typing task in each seat configuration and rested for at least 5 min before the next configuration [17,24]. The order of seat configurations was based on the balanced Latin square design [25]. In each trial, the EMG signals were collected throughout the trial, the body part discomfort (BPD) scale was administered at baseline and at 10 min intervals, and the seat configuration evaluation scale was administered at the end. Video recordings were acquired from the right side of the subjects in each trial.

### 2.3. Data Collection

#### 2.3.1. EMG Signals

A total of 14 muscles were studied: bilateral trapezius (Tr), lumbar erector spinae (LES), lumbar multifidus (LM), external oblique (EO), rectus femoris (RF), gastrocnemius medial (GM), and gastrocnemius lateralis (GL). The EMG signals were recorded using the Noraxon Desktop DTS system and Noraxon MR3 3.10.64 software (Noraxon USA Inc. in Scottsdale, AZ, USA), with a sampling frequency of 1500 Hz and low-pass filtering of 500 Hz. Before the electrode placements, the skin at the target areas was prepared by shaving (if necessary), followed by rubbing and cleaning with alcohol pads [26]. Bipolar disposable Ag/AgCl surface electrodes were placed, as detailed in Table 2, after the skin was dry. The electrodes were placed parallel to the muscle fibers, with a center-to-center distance of approximately 2–3 cm. Tape was used to ensure good fixation of the electrodes and sensors, and elastic bandages were applied as necessary [26].

Prior to the experiment, 4–5 s of maximum voluntary contraction (MVC) was performed twice for each muscle [27]. A rest period of at least 1 min was provided between each MVC trial [28]. Table 2 shows the MVC trials, during which the subjects were provided with manual resistance and frequent verbal encouragement from the researcher. The subjects were instructed to practice the MVC tasks before the formal trials.

**Table 2:** Electrode placements and MVC trials for each muscle (MVC, maximum voluntary contraction; Tr, trapezius; LES, lumbar erector spinae; LM, lumbar multifidus; EO, external oblique; RF, rectus femoris; GM, gastrocnemius medial; GL, gastrocnemius lateralis; C7, the seventh cervical vertebra; L1/L2/L5, the first/second/fifth lumbar vertebra).

Muscle	Electrode Placement	MVC Trial
Tr	2 cm lateral to the midpoint of the line between C7 and the acromion [29]	Subjects shrugged the shoulders in a sitting position [30].
LES	3 cm lateral to L1 [31]	Subjects extended the back in a prone position with the legs secured, the trunk suspended, and the arms crossed over the chest [32].
LM	L5 level, parallel to the line between the posterior superior iliac spine and the L1–L2 interspinous space [33]	
EO	15 cm lateral to the umbilicus [34], aligned at an 80-degree angle to the horizontal [35]	Subjects performed trunk flexion as well as left and right twists in a supine trunk-lifted position, with the feet secured and the knees flexed [36,37].
RF	The midpoint of the line between the anterior superior iliac spine and the superior part of the patella [38]	Subjects performed hip flexion and knee extension simultaneously in a sitting position [39].
GM	The midpoint of the line between the medial side of the popliteal fossa and the medial side of the Achilles tendon insertion [40]	Subjects performed single-leg toe standing, with balanced support provided (if necessary) [41,42].
GL	1/3 of the line between the head of the fibula and the heel [38]	

#### 2.3.2. BPD Scale

Adapted from a previous study [43], the BPD scale was a six-point scale (0 = no discomfort, 5 = extreme discomfort) [44] and included 10 body parts (neck, bilateral shoulders, upper back, lower back, buttocks, bilateral thighs, and bilateral lower legs).

#### 2.3.3. Seat Configuration Evaluation Scale

Each seat configuration was evaluated across two dimensions on seven-point scales: the degree of liking (1 = extremely dislike, 7 = extremely like) and the willingness to use (1 = extremely unwilling, 7 = extremely willing).

### 2.4. Data Processing

For the EMG signals collected during each 30 min task trial, 30 s segments were selected at 5 min intervals starting after 3 min, resulting in six segments per trial. The time-interval-based method was adapted from that used in the previous study [45]. The middle 80% of the EMG signals collected during each MVC trial were selected. All selected EMG signals were filtered with a 4th-order band-pass Butterworth filter (30–500 Hz), in which the 30 Hz cutoff was used to reduce electrocardiogram contamination [46,47]. Then, the signals were baseline corrected, full-wave rectified [48], smoothed using a 0.1 s moving window to obtain the root mean square (RMS) envelopes, and normalized to the maximum RMS value of the corresponding MVC trials [49]. Finally, the mean of the normalized RMS envelope was calculated [50], yielding six mean values for each muscle in each seat configuration level. Outliers were identified and removed based on experimental records (field observations, photos, and videos) and visual inspection [51] of the filtered signals. The data from one subject was excluded, since more than 20% of this data was outliers. After this exclusion, outliers accounted for less than 6% of the total dataset. Following the removal of the outliers, the mean of the remaining data (from the original six values) was calculated for each muscle in each level and expressed as %MVC for statistical analysis. When there was no remaining data, the corresponding %MVC value was replaced by the mean of %MVC values from all other subjects. The replaced values accounted for less than 2% of the entire dataset.

For the BPD score, the data collected from one subject at a specific time point and in a certain seat configuration level was missing and was replaced by the median of available values from all other subjects under the same condition. The overall body discomfort (OBD) was calculated as the mean discomfort of 10 body parts [52]. The BPD frequency (BPDF) was calculated as the frequency of non-zero scores, and the BPD severity (BPDS) was calculated as the mean of non-zero scores [53]. When there were no non-zero scores, both the BPDF and BPDS were 0. Referring to a previous study [54], the BPDF and BPDS were calculated for the overall body and three body segments: the neck–shoulder (neck and bilateral shoulders), the back (upper and lower back), and the lower limb (buttocks, bilateral thighs, and bilateral lower legs). The mean values of the BPD score, BPDF, and BPDS at four time points were calculated for statistical analysis.

### 2.5. Statistical Analysis

All data from one subject was excluded owing to failure to follow experimental requirements, and the EMG data from one subject was excluded owing to excessive outliers. The scale data from 16 subjects and the EMG data from 15 subjects were statistically analyzed. A one-way repeated-measures analysis of variance was performed to determine significant differences in seat configurations, and the least significant difference (LSD) test was used for pairwise comparisons. Data analysis was performed in SPSS 25, with a significance level of 0.05. The effect size is small, medium, or large when the partial η^2^ value is no less than 0.01, 0.059, or 0.138, respectively [55].

## 3. Results

### 3.1. Muscle Activity

Left LES (F = 3.073, *p* = 0.023, partial η^2^ = 0.180) and right LES (F = 9.392, *p* = 0.001, partial η^2^ = 0.774) muscle activity differed significantly across seat configurations, with large effect sizes. No significant differences in regards to other muscle activity were observed across seat configurations.

**Figure 4:**
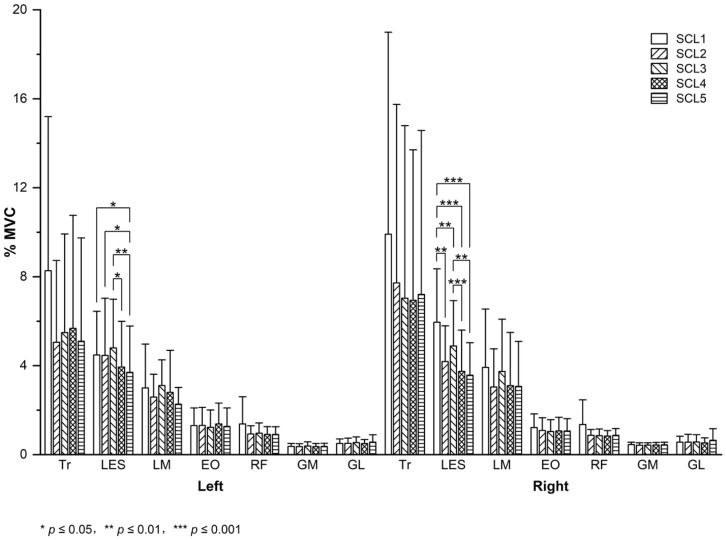
Mean (M) and standard deviation (SD) of bilateral muscle activity of seven muscles in each seat configuration (MVC, maximum voluntary contraction; Tr, trapezius; LES, lumbar erector spinae; LM, lumbar multifidus; EO, external oblique; RF, rectus femoris; GM, gastrocnemius medial; GL, gastrocnemius lateralis; SCL1/SCL2/SCL3/SCL4/SCL5, seat configuration level 1/2/3/4/5).

The LSD results are shown in Figure 4. For left LES muscle activity, significant differences were observed between SCL1 and SCL5 (*p* = 0.028), between SCL2 and SCL5 (*p* = 0.023), between SCL3 and SCL4 (*p* = 0.040), and between SCL3 and SCL5 (*p* = 0.002). Left LES muscle activity in SCL5 decreased by 17.42%, 17.03%, and 22.93% compared with that in SCL1, SCL2, and SCL3, respectively. Left LES muscle activity in SCL4 decreased by 17.94% compared with that in SCL3. For right LES muscle activity, significant differences were observed between SCL1 and SCL2 (*p* = 0.003), between SCL1 and SCL3 (*p* = 0.003), between SCL1 and SCL4 (*p* < 0.001), between SCL1 and SCL5 (*p* = 0.001), between SCL3 and SCL4 (*p* < 0.001), and between SCL3 and SCL5 (*p* = 0.008). Right LES muscle activity in SCL2, SCL3, SCL4, and SCL5 decreased by 29.72%, 17.91%, 37.08%, and 39.99% compared with that in SCL1, respectively. Right LES muscle activity in SCL4 and SCL5 decreased by 23.35% and 26.89% compared with that in SCL3, respectively.

### 3.2. Perceived Discomfort

Left shoulder discomfort (F = 4.152, *p* = 0.005, partial η^2^ = 0.217), upper back discomfort (F = 3.631, *p* = 0.037, partial η^2^ = 0.548), lower back discomfort (F = 5.420, *p* = 0.001, partial η^2^ = 0.265), BPDF back (F = 9.440, *p* < 0.001, partial η^2^ = 0.386), and BPDS back (F = 3.586, *p* = 0.011, partial η^2^ = 0.193) differed significantly across seat configurations, with large effect sizes. No significant differences in other BPD scores, BPDF, and BPDS were observed across seat configurations.

**Figure 5:**
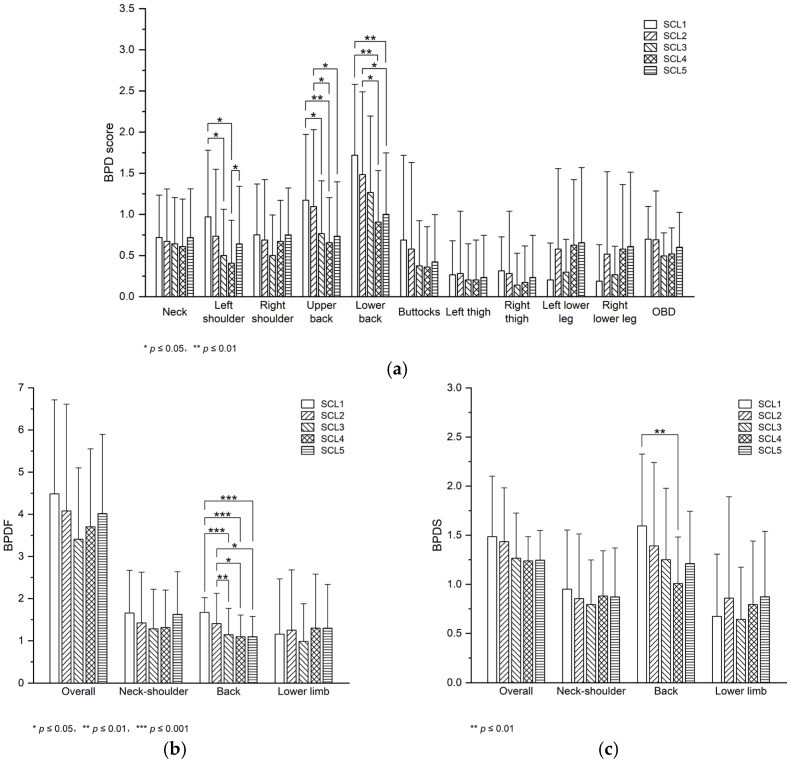
Mean (M) and standard deviation (SD) of perceived discomfort ((**a**) BPD score, (**b**) BPDF, (**c**) BPDS) in each seat configuration (BPD, body part discomfort; OBD, overall body discomfort; BPDF, body part discomfort frequency; BPDS, body part discomfort severity; SCL1/SCL2/SCL3/SCL4/SCL5, seat configuration level 1/2/3/4/5).

The LSD results are shown in Figure 5. For left shoulder discomfort, significant differences were observed between SCL1 and SCL3 (*p* = 0.013), between SCL1 and SCL4 (*p* = 0.012), and between SCL4 and SCL5 (*p* = 0.027). Left shoulder discomfort in SCL3 and SCL4 decreased by 48.40% and 58.10% compared with that in SCL1, respectively. Left shoulder discomfort in SCL4 decreased by 36.66% compared with that in SCL5. For upper back discomfort, significant differences were observed between SCL1 and SCL3 (*p* = 0.025), between SCL1 and SCL4 (*p* = 0.007), between SCL2 and SCL4 (*p* = 0.029), and between SCL2 and SCL5 (*p* = 0.036). Upper back discomfort in SCL3 and SCL4 decreased by 34.64% and 44.03% compared with that in SCL1, respectively. Upper back discomfort in SCL4 and SCL5 decreased by 40.04% and 32.91% compared with that in SCL2, respectively. For lower back discomfort, significant differences were observed between SCL1 and SCL4 (*p* = 0.002), between SCL1 and SCL5 (*p* = 0.007), between SCL2 and SCL4 (*p* = 0.033), and between SCL2 and SCL5 (*p* = 0.028). Lower back discomfort in SCL4 and SCL5 decreased by 47.29% and 41.83% compared with that in SCL1, respectively. Lower back discomfort in SCL4 and SCL5 decreased by 38.95% and 32.61% compared with that in SCL2, respectively.

For BPDF back, significant differences were observed between SCL1 and SCL3 (*p* = 0.001), between SCL1 and SCL4 (*p* < 0.001), between SCL1 and SCL5 (*p* < 0.001), between SCL2 and SCL3 (*p* = 0.010), between SCL2 and SCL4 (*p* = 0.024), and between SCL2 and SCL5 (*p* = 0.024). BPDF back in SCL3, SCL4, and SCL5 decreased by 31.76%, 34.57%, and 34.57% compared with that in SCL1, respectively. BPDF back in SCL3, SCL4, and SCL5 decreased by 18.85%, 22.19%, and 22.19% compared with that in SCL2, respectively. For BPDS back, significant differences were observed between SCL1 and SCL4 (*p* = 0.002). BPDS back in SCL4 decreased by 36.76% compared with that in SCL1.

### 3.3. Seat Configuration Evaluation

No significant differences in the degree of liking and the willingness to use were observed across seat configurations. The degree-of-liking score was the highest in SCL4 (M = 4.500, SD = 1.095) and the lowest in SCL1 (M = 4.000, SD = 1.317). The willingness-to-use score was the highest in SCL4 (M = 4.250, SD = 1.528) and the lowest in SCL1 (M = 3.875, SD = 1.628) and SCL5 (M = 3.875, SD = 1.708).

## 4. Discussion

Our study found that the unsupported kneeling posture, compared with the unsupported traditional sitting posture, resulted in significantly less LES muscle activity and perceived discomfort (shoulder discomfort, upper and lower back discomfort, BPDF back, and BPDS back). Among the four angle combinations of the kneeling chair, SCL4 resulted in a significant decrease in both LES muscle activity and perceived discomfort (shoulder discomfort, upper and lower back discomfort, and BPDF back). For comparison, Table 3 summarizes the significant differences observed in our study and the results of related indicators from previous studies and presents the corresponding methodologies employed. Given that no previous studies have focused on angle combinations, we compared our findings with those from prior work on individual angles, namely, the seat pan angle and the knee rest angle. Since Soderberg et al. [14] did not specify the knee rest angle in their study on the seat pan angle, the study by Tang et al. [20] that investigated both angles was used for comparison. The angles explored were knee rest angles of 20, 30, and 40 degrees (KA20, KA30, and KA40), with SA20; seat pan angles of 10, 20, and 30 degrees (SA10, SA20, and SA30), with KA30. Namely, the angle combinations of the kneeling configurations were SA10–KA30, SA20–KA30, SA30–KA30, SA20–KA20, and SA20–KA40.

As shown in Table 3, our findings on LES muscle activity for the comparison between the kneeling configuration and the traditional sitting configuration align with those of Soderberg et al. [14]. Although their study found no significant difference in subjective comfort, our results are consistent with the non-significant trend that they reported. In terms of angle combinations, our study found that LES muscle activity in SCL5 (SA20–KA35) was significantly less than that in SCL3 (SA10–KA35). This aligns with the finding of Tang et al. [20] that, with KA30 (close to KA35), LES muscle activity in SA20 was significantly less than that in SA10. In addition, Tang et al. [20] reported a non-significant trend that with SA20, LES muscle activity in KA20 was less than that in KA30. Two findings from their study may suggest a trend towards less LES muscle activity in the SA20–KA20 combination than in the SA10–KA30 (close to SA10–KA35) combination, though no direct comparison was made between the two combinations. In our study, LES muscle activity in SCL4 (SA20–KA20) was significantly less than that in SCL3 (SA10–KA35), which is consistent with this inferred trend. Further comparisons regarding muscle activity and subjective (dis)comfort are difficult to conduct, owing to limited findings from previous studies.

Additionally, there are discrepancies between our findings and those of prior studies. We observed no significant difference in Tr muscle activity between the traditional sitting posture and the kneeling posture, while Soderberg et al. [14] reported significant results. One possible explanation for the discrepancy is the difference in neck posture due to the screen placement during the experimental task. The subjects in our study likely maintained a more horizontal line of sight and a more upright head position compared with those in their study. Moreover, our findings on LES and gastrocnemius muscle activity, upper and lower back discomfort contradict those of Wang et al. [15] in the comparison between the kneeling configuration and the traditional sitting configuration. Our results were obtained from a 30 min typing task performed by healthy adults, while their results came from a 60 min handwriting task performed by healthy children. Variations in experimental tasks, experimental durations, subject characteristics, and subjects’ familiarity with or adaptation to the kneeling posture, individually or in combination, may lead to these inconsistent findings. Furthermore, for the kneeling configurations, our results are consistent with those of Tang et al. [20] regarding LES muscle activity, but inconsistent with their findings regarding gastrocnemius muscle activity. The discrepancy in regards to gastrocnemius muscle activity may mainly arise from postural constraints on the leg imposed by the kneeling posture. In terms of LES muscle activity, our results are consistent with those of Tang et al. [20] but inconsistent with those of Wang et al. [15]. This discrepancy may be primarily due to familiarity with or adaptation to the kneeling posture, since the two prior studies adopted similar experimental designs except for the seat configuration conditions. And we note that though significant results in terms of perceived discomfort showed a clear tendency towards specific seat configurations, no significant findings were observed in seat configuration evaluation (i.e., the degree of liking and the willingness to use). One possible explanation is that this evaluation is a more comprehensive measure, as it is likely influenced by multiple factors, including (dis)comfort, adaptability, aesthetics, etc.

One limitation of this study is the single subject group, since young healthy adults represent only a portion of the real-world users. Future studies should investigate optimal angle combinations of the kneeling chair for user groups with different characteristics, such as age and health status. Additionally, only the typing task was included in this study. Since users perform various tasks when using the kneeling chair in practice, different types of tasks should be considered in the future. Furthermore, the muscles selected in this study were based on the following criteria: measurability via EMG technology, extensive investigation in previous similar studies, and representativeness across different body parts. However, two limitations exist: one is the lack of a more thorough analysis of the kneeling posture in the field of biomechanics, and the other is the constraints of the adopted technology, including the difficulty in recording the activity of deep muscles. Thus, the muscles measured in this study do not fully represent the core muscles involved in maintaining the kneeling posture. Future research should refine the muscle selection and adopt more suitable technologies to measure the activity of core muscles. In addition to EMG signals, other objective data (e.g., surface pressure and joint angles) could be collected in the future to conduct a more comprehensive assessment of the kneeling chair angle combinations. In summary, our results should be interpreted with caution due to these limitations, and further studies should be conducted to enhance the generalizability of the conclusions.

## 5. Conclusions

This study assessed an unsupported traditional sitting configuration and four unsupported kneeling configurations with different angle combinations by measuring EMG signals, BPD scores, and seat configuration evaluation scores. Based on significant differences in LES muscle activity and perceived discomfort, the unsupported kneeling configuration was found to be preferable. And within the scope of this study, the optimal angle combination of the kneeling chair was identified as both the seat pan angle and the knee rest angle being set at 20 degrees. Our results can serve as a reference for kneeling chair designers and researchers.

## Figures and Tables

**Table 1:** Comparisons between the kneeling chair and the traditional chair obtained from previous studies in regards to muscle activity and subjective (dis)comfort.

Indicator	Study	The Kneeling Chair vs. the Traditional Chair
Muscle activity	Bennett et al. [9]	No significant difference in erector spinae muscle activity.
	Soderberg et al. [14]	Significantly less trapezius and erector spinae muscle activity using the kneeling chair.
	Cram et al. [16]	Significantly greater lumbar paraspinal muscle activity using the kneeling chair; no significant differences in cervical and thoracic paraspinal muscle activity.
	Lander et al. [17]	Significantly greater cervical paraspinal muscle activity using the kneeling chair; no significant difference in lumbar paraspinal muscle activity.
	Wang et al. [15]	Significantly greater erector spinae and gastrocnemius muscle activity using the kneeling chair.
Subjective (dis)comfort	Lander et al. [17]	A non-significant preference for the traditional chair in terms of overall and lower back comfort.
	Soderberg et al. [14]	A non-significant preference for the kneeling chair in terms of comfort.
	Wang et al. [15]	A non-significant trend towards greater comfort in the overall body, upper back, lower back, buttocks, thighs, and lower legs using the traditional chair; a non-significant trend towards greater neck comfort using the kneeling chair.
	Bishu et al. [18]	A non-significant trend towards greater discomfort in the upper, middle, and lower back using the kneeling chair.
	Bridger et al. [19]	Significantly greater comfort using the kneeling chair in combination with both horizontal and inclined work surfaces.

**Table 3:** Methodologies and results of this study and related previous studies (Tr, trapezius; LES, lumbar erector spinae; BPDF, body part discomfort frequency; BPDS, body part discomfort severity; M, mean; SCL1/SCL2/SCL3/SCL4/SCL5, seat configuration level 1/2/3/4/5; SA10/SA20/SA30, seat pan angle of 10/20/30 degrees; KA20/KA30/KA35/KA40, knee rest angle of 20/30/35/40 degrees).

Study	Subjects	Unsupported Seat Configurations	Experimental Task	Results
This study	Sample size: 17 healthy subjects (8 females and 9 males) Age (years): 19–30, M = 22.82	Traditional sitting configuration (SCL1); kneeling configurations: SA10–KA20 (SCL2), SA10–KA35 (SCL3), SA20–KA20 (SCL4), and SA20–KA35 (SCL5) combinations.	Typing for 30 min	LES muscle activity, shoulder discomfort, upper and lower back discomfort, BPDF back, BPDS back: kneeling < traditional (significant). BPDF back: SCL2 > SCL3 (significant). Upper and lower back discomfort, BPDF back: SCL2 > SCL4 (significant). LES muscle activity, upper and lower back discomfort, BPDF back: SCL2 > SCL5 (significant). LES muscle activity: SCL3 > SCL4 (significant); SCL3 > SCL5 (significant). Shoulder discomfort: SCL4 < SCL5 (significant).
Tang et al. [20]	Sample size: 6 healthy subjects (6 females) Age (years): 7–9	Kneeling configurations: SA10–KA30, SA20–KA30, SA30–KA30, SA20–KA20, and SA20–KA40 combinations.	Handwriting on paper for 60 min	LES muscle activity: SA10–KA30 > SA20–KA30 > SA30–KA30 (significant); SA20–KA30 > SA20–KA20 (non-significant). Gastrocnemius muscle activity: SA10–KA30 < SA20–KA30 < SA30–KA30 (significant); SA20–KA30 < SA20–KA40 < SA20–KA20 (significant).
Wang et al. [15]	Sample size: 6 healthy subjects (6 females) Age (years): M = 8	Traditional sitting configuration; kneeling configuration: SA10–KA30 combination.	Handwriting on paper for 60 min	LES and gastrocnemius muscle activity: kneeling > traditional (significant). Upper and lower back comfort: kneeling < traditional (non-significant).
Soderberg et al. [14]	Sample size: 20 healthy subjects (10 females and 10 males) Age (years): 22–33, M = 24.4	Traditional sitting configuration; kneeling configurations: SA10 and SA20, with an unmentioned knee rest angle.	Minimal-typing computer interaction for 15 min (20 subjects) or 30 min (10 subjects)	Tr and LES muscle activity: kneeling < traditional (significant); SA10 > SA20 (significant). Subjective comfort: kneeling > traditional (non-significant); SA10 < SA20 (non-significant).

## Data Availability

The data supporting the findings of this study are available from the corresponding author upon reasonable request.

## Abbreviations

The following abbreviations are used in this manuscript:

EMG	surface electromyography
BPD	body part discomfort
LES	lumbar erector spinae
BMI	body mass index
SCL1/SCL2/SCL3/SCL4/SCL5	seat configuration level 1/2/3/4/5
SA10/SA20/SA30	seat pan angle of 10/20/30 degrees
KA20/KA30/KA35/KA40	knee rest angle of 20/30/35/40 degrees
Tr	trapezius
LM	lumbar multifidus
EO	external oblique
RF	rectus femoris
GM	gastrocnemius medial
GL	gastrocnemius lateralis
MVC	maximum voluntary contraction
C7	the seventh cervical vertebra
L1/L2/L5	the first/second/fifth lumbar vertebra
RMS	root mean square
OBD	overall body discomfort
BPDF	body part discomfort frequency
BPDS	body part discomfort severity
LSD	the least significant difference
M	mean
SD	standard deviation
